# Dampening of Positive Affect Serves an Emotional Contrast Avoidance Function: Preliminary Evidence From an Adult Community Sample

**DOI:** 10.1002/jclp.70060

**Published:** 2025-11-10

**Authors:** Liesbeth Bogaert, Miguel A. Segura-Vargas, Barnaby D. Dunn, David J. Hallford, Michelle G. Newman, Filip Raes

**Affiliations:** 1 Faculty of Psychology and Educational Sciences, KU Leuven, Leuven, Belgium; 2 Mood Disorders Centre, University of Exeter, Exeter, UK; 3 School of Psychology, Deakin University, Geelong, Victoria, Australia; 4 Department of Psychology, The Pennsylvania State University, University Park, Pennsylvania, USA

**Keywords:** dampening, emotion regulation, emotional contrast avoidance

## Abstract

Dampening of positive affect (PA) constitutes a transdiagnostic risk and maintenance factor for affective dysregulation in various psychopathologies, including depression. However, the motives underlying this PA downregulation strategy remain unclear, even though they may be highly relevant for improving traditional psychological treatments. This study examined whether avoidance of negative emotional contrasts (NECs) and diminished preference for positive emotions were predictive of dampening. The latter was operationalised as low pro- and high contra-hedonic emotion regulation (ER) goal endorsement. An adult community sample (*N* = 159) completed an online survey, and multiple linear regressions were conducted to examine the predictive validity of both factors, after controlling for age, gender, and repetitive negative thinking (RNT). Higher levels of NEC avoidance and higher contra-hedonic ER goal endorsement were consistently found to uniquely predict concurrent dampening levels, above and beyond age, gender and RNT. Crucially, inclusion of both factors in the same regression model still yielded evidence for the unique predictive validity of NEC avoidance. Findings support the possibility that dampening is motivated by NEC avoidance rather than solely by emotional preferences. Study limitations are noted, and implications for future research and clinical practice are discussed.

Dampening refers to a response style toward positive affect (PA) encompassing mental strategies that downregulate the intensity and/or frequency of PA states (e.g., “This positive feeling won’t last”; Feldman et al. 2008). The majority of research on dampening so far has been situated in the field of depression, of which blunted PA levels or anhedonia are central features (American Psychiatric Association 2013; Forbes et al. 2010; Kendall et al. 2015; Morris et al. 2009; Rackoff and Newman 2020). More specifically, in this field, dampening of PA represents a distinct emotion regulation (ER) strategy contributing to symptomatology, among various other ER strategies implicated in its aetiology and maintenance (Everaert et al. 2022; Joormann and Gotlib 2010; Joormann and Stanton 2016; Joormann and Vanderlind 2014; Vanderlind et al. 2020).

A substantial body of empirical evidence indeed supports the role of dampening as a vulnerability and/or maintenance factor of depressive symptomatology. Meta-analytic findings indicated a bidirectional positive relationship between trait dampening and depressive symptoms (Bean et al. 2022), challenging the notion that dampening is merely a consequence of symptomatology (Vanderlind et al. 2020). Trait dampening also predicted depressive symptoms, beyond repetitive negative thinking, a well-established risk factor (McEvoy et al. 2018; Raes et al. 2012; Raes et al. 2014). In daily life, time-series studies showed that higher state dampening predicted higher depressive symptoms and reduced PA (Li et al. 2017; Vanderlind et al. 2022).

However, although the link between dampening and depressive symptoms is well-established, evidence increasingly indicates that its effects clearly extend beyond depression, highlighting its potential as a transdiagnostic ER strategy. This was demonstrated by dampening’s positive association with various other forms of psychopathology, including posttraumatic stress (Boelen 2021; Kiefer et al. 2023; Wolkenstein et al. 2022), prolonged grief (Lenferink et al. 2018), psychosis (Damme et al. 2022), eating difficulties (Dworschak et al. 2023), and various anxiety-related conditions (Abasi et al. 2023; Baik and Newman 2023, 2025; Buhk et al. 2020; Eisner et al. 2009; Everaert et al. 2020). Experimental manipulations in the lab and in naturalistic settings further demonstrated that dampening, irrespective of psychopathology, causally altered affective states, with dampening thoughts increasing sadness and decreasing happiness (Burr et al. 2017; Dunn et al. 2018; Yilmaz et al. 2019). Therefore, gaining insight into dampening and its function may in fact help identify a critical transdiagnostic target, relevant across a broad range of psychopathologies.

To advance the current understanding of dampening of PA and ultimately inform the development and optimization of psychological interventions, this study examined two proposed perspectives on the function of dampening as a transdiagnostic process. The first perspective, inspired by the Contrast Avoidance Model (CAM; Newman and Llera 2011), states that the deployment of dampening may follow from the motive to avoid negative emotional contrasts (NECs) or sharp increases/decreases in negative/positive affect (NA/PA; Bean et al. 2022; Buhk et al. 2020; Dunn and Roberts 2016; Malivoire et al. 2022; Vanderlind et al. 2022). Originally, the CAM was developed to explain the NECs avoidance function of worry in generalized anxiety disorder (GAD; Newman et al. 2023). By increasing and sustaining NA, as a form of homeostasis, worry enables an individual to avoid NECs (Newman and Llera 2011; Newman et al. 2013). More recent evidence suggests that rumination may also serve a NEC avoidance function in depression by nourishing NA (Baik and Newman 2023; Jamil and Llera 2021; Kim and Newman 2022; Newman et al. 2023).

Analogously, dampening may be another pathway to avoid NECs, through the downregulation of PA experiences (Baik and Newman 2023; Bean et al. 2022; Buhk et al. 2020; Dunn and Roberts 2016; Vanderlind et al. 2022). Dampening thoughts such as “This positive event is just an exception which won’t happen again” or “This positive feeling won’t last” may instantaneously downregulate glimmerings of PA initiated by a positive event (e.g., positive feedback on a project at work). In this way, these thoughts may pre-empt an affective uplift, possibly to avoid the NEC that occurs when PA returns to the individual’s baseline somber state (i.e., sharp downward PA shift). Moreover, PA dampening induction has been found to simultaneously increase NA (Burr et al. 2017; Dunn et al. 2018). Thus, dampening may even serve a NEC avoidance function by elevating NA, in a manner similar to worry and rumination. Taken together, dampening may be a strategy to maintain homeostasis to ultimately avoid NECs, at the cost of not having affective uplifts at all (Dunn and Roberts 2016).

Recent empirical evidence in the GAD field showed that dampening may indeed be linked to such a motive to avoid NECs (Baik and Newman 2023; Malivoire et al. 2022). The current study aimed to extend existing evidence in two ways: by addressing a methodological limitation through improved operationalization of the concepts of interest (see Measures), and by ruling out the potential confounding effect of dampening’s association with repetitive negative thinking (RNT), a well-established transdiagnostic ER strategy (Moulds and McEvoy 2025). As noted earlier, prior research showed that worry and rumination—prototypical examples of RNT—could serve a NEC avoidance function (Baik and Newman 2023; Jamil and Llera 2021; Kim and Newman 2022; Newman et al. 2019; Newman et al. 2023; Newman et al. 2022), and RNT was found to be positively associated with dampening (McEvoy et al. 2018). Therefore, this study additionally tested whether NEC avoidance also uniquely explained variance in concurrent dampening, which could not be explained by its shared link with RNT.

The second proposed perspective on which this study focuses, is rooted in the instrumental model of ER (Tamir 2009; Tamir 2016). In essence, this model states that emotional preferences partially steer ER strategy use, or put differently, that individuals use those ER strategies that are congruent with their emotion preference (Tamir 2009; Tamir 2016). So far, the majority of empirical studies testing this model have focused on negative ER strategies, whereas associations between emotional preferences and positive ER strategies were largely neglected (Vanderlind et al. 2020).

Therefore, the current study examined the relation between diminished preference for positive emotions, and dampening as an emotion preference-congruent ER strategy (Vanderlind et al. 2020). To this end, diminished preference for positive emotions was operationalised by the level of endorsement in hedonic ER goals. Specifically, following the classification of Eldesouky and English (2019), contra-hedonic ER goal endorsement refers to the desire to increase/maintain NA and decrease PA. Pro-hedonic ER goal endorsement involves the desire to increase/maintain PA and decrease NA (Eldesouky and English 2019). Taken together, the tendency to use dampening might be driven by high endorsement of contra-hedonic and/or low endorsement of pro-hedonic ER goals, as they presumably reflect an underlying diminished preference for positive emotions.

Similar to the first perspective, the role of RNT should be considered since RNT is likely to be associated with high endorsement of contra-hedonic and/or low endorsement of pro-hedonic ER goals. For instance, depressed individuals have been found to actively maintain sadness (Millgram et al. 2019), which may be easily achieved via RNT (Stefanovic et al. 2022). In other words, to capture the variance of dampening explained by the mere diminished preference for PA, the association between dampening and RNT (and NEC avoidance) should be controlled for.

In short, the first study objective was to examine whether higher levels of avoidance of NECs were predictive of higher concurrent dampening, above and beyond age, gender and RNT (H1). In addition, as a second study objective, the predictive validity of the endorsement of contra- (H2a) and pro-hedonic (H2b) ER goals was investigated, after controlling for covariates age, gender, RNT and avoidance of NECs. That is, higher levels of contra-hedonic goals (H2a) and lower levels of pro-hedonic goals (H2b) were expected to be predictive of higher levels of concurrent dampening. Importantly, levels of psychopathology (e.g., depressive symptoms, PTSS, or anxiety symptoms) were not included as covariates or confounding factors, as they cannot theoretically explain (and may even remove crucial variance to understand) underlying motivational processes of dampening as a transdiagnostic strategy.

## 1 | Methods

### 1.1 | Participant Characteristics

A total of 173 English-speaking adults enrolled in this online study, which took place in February 2023. Participants with incomplete survey responses (*n* = 5) were excluded, as well as those whose survey completion time was defined as outliers based on the interquartile range (IQR) rule (i.e., below Q1 (or Pc 25) − 1.5 × IQR or above Q3 (or Pc 75) + 1.5 × IQR; *n* = 9). The final sample consisted of 159 participants (*M*_age_ = 35.99, SD_age_ = 12.39, range 18–73; 49% female, 51% male). Self-identified ethnicity was distributed as follows: 74.21% for White/Caucasian, 12.58% for African, 5.03% for Mixed, 1.26% for both Black/African American and East Asian, 2.52% for South Asian, and 0.63% for all the other categories (Latino/Hispanic, White Mexican, Black/British, Native American or Alaskan Native and other).

### 1.2 | Sampling and Data Collection Procedure

After receiving ethical approval (Social and Societal Ethics Committee of KU Leuven) and preregistering the study (Open Science Framework; https://osf.io/3rh7a), recruitment of participants took place via Prolific, an online data collection platform (www.prolific.co). Prolific has been found to provide high data quality compared to other platforms commonly used in behavioral research (Peer et al. 2022). Eligible participants were native English-speaking individuals (18–80 years) with a Prolific account. Stratified sampling allowed us to collect data from a balanced sample in terms of gender. No other eligibility criteria were applied. After providing informed consent and basic demographic information (age, gender, and ethnicity), participants were invited to complete a series of self-report scales via a Qualtrics survey (www.Qualtrics.com). After study completion, participants obtained remuneration via their Prolific account according to the recommended payment principles of Prolific (£9.00/$12.00 per hour). For the estimated duration for survey completion of 8 min, participants obtained £1.20/$1.43.

### 1.3 | Sample Size Planning

Sample size planning relied on an a priori power analysis conducted in G*Power (version 3.1.9.4), which yielded a required sample size of 157 participants. This sample size allowed detection of a small-to-moderate effect (Cohen’s *f*^2^ = 0.085) via linear multiple regression models (*α* = 0.05, *β* = 0.80 power) with five predictors (avoidance of NECs, contra-/pro-hedonic ER goals, RNT, gender, and age). Close monitoring of data collection for drop-outs was required for the process of manually granting participants their remuneration, which obviated the need for oversampling strategies.

### 1.4 | Measures

The 8-item dampening subscale of the English Responses to Positive Affect scale (RPA-d; Feldman et al. 2008) measured participants’ general tendency to engage in dampening. The RPA-d was included to allow for comparison with prior findings, given its use in earlier studies examining the NEC function of dampening. Participants rated items that reflected dampening thoughts (e.g., “When you feel happy, how often do you think about things that could go wrong?”) on a 4-point rating scale from 1 (*almost never*) to 4 (*almost always*). Internal consistency in the current sample was good (*α* = 0.84).

To further assess dampening, participants also completed the newly developed Leuven Exeter Dampening Scale (LEDS; Bogaert et al. 2025). The LEDS was designed as a more comprehensive alternative to existing measures, aiming to capture the nuanced clinical presentation of dampening more effectively. Compared to the RPA-d, it incorporates additional commonly observed dampening thoughts, such as “I would have enjoyed this more previously” or “There is no point to feeling good.” A full list of LEDS items, along with the class of dampening thoughts they represent and evidence supporting the presumed one-factor solution (Bogaert et al. 2025), is provided in the Supporting Information (Table S1). Because the aim was to include the LEDS to strengthen construct coverage and to determine if the same effect would be replicated, we did not apply multiple comparison corrections. The LEDS consists of 13 items, which are rated on a 5-point scale ranging from 1 (*almost never*) to 5 (*almost always*). In the current sample, internal consistency was excellent (*α* = 0.93). Higher scores on both dampening scales represent higher levels of dampening.

The discomfort with emotional shifts subscale of the Contrast Avoidance Questionnaire for General Emotions (CAQ-GE; Llera and Newman 2017) assessed the extent to which individuals felt uncomfortable with sharp emotional shifts, as a proxy for emotional contrast avoidance. Participants rated 7 items (e.g., “I feel uneasy with emotional changes”) on a 5-point scale going from 1 (*Not at all true*) to 5 (*Absolutely true*) with higher scores reflecting higher emotional discomfort with emotional shifts. Excellent internal consistency was found in the current sample (*α* = 0.92). In contrast with the approach adopted in Malivoire et al. (2022), only the discomfort domain-specific factor of the CAQ-GE (Llera and Newman 2017) was used to measure NEC avoidance. We adopted this approach to avoid overlap between the items used to measure NEC avoidance and dampening. The use of the Avoidance domain-specific factor (cf. Malivoire et al. 2022) would have implied such conceptual overlap as items of this scale allude to dampening thoughts. Importantly, recent evidence supported the use of the discomfort domain-specific factor on its own (Rogers et al. 2023).

The Emotional Regulation Goals Scale (ERGS; Eldesouky and English 2019) measured individuals’ general endorsement of ER goals. Via 18 items that were rated on a 7-point scale from 1 (*never*) to 5 (*always*). This scale covers five subscales: pro-hedonic (3 items; e.g., “to feel less negative emotion (e.g., anger, sadness)”), contra-hedonic (3 items; e.g., “to feel more negative emotion (e.g., anger, sadness)”), performance (3 items; e.g., “to avoid being distracted by how you’re feeling?”), prosocial (5 items; e.g., “to make someone else feel good?”) and impression management goals (4 items; e.g., “to avoid making an unfavorable impression on others?”). Internal consistencies for all subscales in the current sample ranged from acceptable-to-good to good-to-excellent (α _pro-hedonic_ = 0.76; α_contra-hedonic_ = 0.78; α_performance_ = 0.83; α_prosocial_ = 0.85; α_impression_ = 0.86). Only results for the pro- and contra-hedonic subscales are reported on. A parallel analysis (Raîche et al. 2013) and subsequent exploratory analysis on the items of the hedonic subscales supported the two-factor structure of the hedonic subscales (i.e., pro- and contra-hedonic; details, see Table S2, Supporting Information).

The core characteristics of the Perseverative Thinking Questionnaire (Ehring et al. 2011) assessed participants’ tendency to engage in RNT. Nine items (e.g., “The same thoughts keep going through my mind again and again”) measuring the core characteristics of RNT, namely repetitiveness, intrusiveness and difficulties with disengagement from thoughts, are rated on a 5-point scale ranging from 0 (*never*) to 4 (*almost always*). Internal consistency in the current sample was excellent (α = 0.95).

### 1.5 | Research Design and Statistical Analysis Plan

As preregistered (https://osf.io/3rh7a), separate multiple linear regressions were conducted on the cross-sectional survey data. Dampening, measured via both dampening scales, systematically acted as dependent variable, and age and gender as covariates. To enable model performance comparison, RNT, avoidance of negative emotional shifts, and contra- and pro-hedonic ER goals (reflecting emotional preference) were added to the model as predictors of dampening in a stepwise way. Model assumptions for linearity, homogeneity, normality of the residuals and multicollinearity (VIF < 5) were visually checked via the “performance” R package (v. 0.10.6; Lüdecke et al. 2021). Finally, all analyses were rerun after outlier removal (*n* = 3) relying on the interquartile range (IRQ) criterion, which defines an outlier as any observation falling below Q1 − 1.5 × IQR or above Q3 + 1.5 × IRQ. Q1 and Q3 refer to the first and third quartile respectively.

Conducted in a post hoc way, all models were rerun without RNT, as controlling for RNT might be overly conservative. Although controlling for RNT ruled out the possibility that avoidance of negative emotional shifts and contra- or pro-hedonic ER goals predicted dampening solely through their shared link with RNT, it may have also removed variance reflecting overlapping underlying motives shared by both ER strategies.

## 2 | Results

### 2.1 | Preliminary Analyses

Tables 1 and 2 present the descriptive statistics and the Pearson’s correlation matrix for all variables respectively. Except for the associations between pro-hedonic and contra-hedonic ER goals, and contra-hedonic ER goals and dampening (measured via both scales; *p*s > 0.12), all outcomes were significantly positively correlated (*ps* < 0.05).

### 2.2 | Main Analyses

Table 3 summarizes the results of all multiple linear regressions performed for dampening as the dependent variable, after pairwise outlier deletion according to the IQR rule. Separate analyses were run for dampening measured via the RPA dampening subscale and the LEDS. No notable model assumption violations were detected. For transparency, results from the preregistered models with RNT (Model 1a, 2a, and 3a) and from the post hoc models without RNT (Model 1b, 2b, and 3b) are reported on in Table 3. Although conclusions are identical, to err on the safe side, results of the most conservative and preregistered models are discussed below.

Model 1a included age, gender and RNT as predictors; model 2a additionally entered NEC avoidance; and model 3a additionally entered contra- and pro-hedonic ER goal endorsement. In line with H1, in model 2a analyses NEC avoidance had a unique predictive value in both the RPA dampening subscale and the LEDS model, above and beyond age, gender and RNT. In the RPA dampening model, NEC avoidance explained an additional 2.4% of variance (a small effect size). In the LEDS dampening model, NEC avoidance explained an additional 7.5% of variance (a small-to-medium effect size).

In support of H2a, contra-hedonic ER goal endorsement was found to be a significant unique predictor of dampening across measures (RPA dampening, LEDS) in model 3a analyses. Only for dampening measured via the LEDS, lower levels of pro-hedonic ER goal endorsement were significantly predictive of higher levels of dampening. For dampening measured via the RPA dampening subscale, no indications were found for the predictive value of pro-hedonic ER goal endorsement. So, the evidence only partly supported H2b, as the predictive value of pro-hedonic ER goal endorsement seemed to depend on the measure of dampening. Hedonic ER goal endorsement in model 3a explained an additional 5.3% of variance (small-to-medium) effect size in the RPA dampening analyses and 14.8% of variance (medium effect size) in the LEDS dampening analyses compared to model 2a. Importantly, the predictive value of NEC avoidance remained significant in model 3a even after controlling for RNT and hedonic ER goal endorsement.

## 3 | Discussion

As a first objective, building on the Contrast Avoidance Model (Newman and Llera 2011), the current study examined the hypothesis that avoidance of NECs had a unique predictive value concerning individuals’ tendency to dampen positivity (e.g., Baik and Newman 2023; Bean et al. 2022; Buhk et al. 2020; Dunn and Roberts 2016; Malivoire et al. 2022; Vanderlind et al. 2022). In support of our hypothesis, results demonstrated the unique value of avoidance of NECs in the prediction of concurrent levels of dampening, above and beyond age, gender and the engagement in RNT.

As to the second objective, the current study tested whether high/low endorsement of contra-/pro-hedonic ER goals (H2a and H2b), as a measure for diminished preference for positive emotions, might be underlying individuals’ dampening response style. In support of H2a, the endorsement of contra-hedonic ER goals was found to be predictive of higher concurrent levels of dampening, above and beyond age, gender, RNT and NEC avoidance. However, H2b was only partially supported. Only for the models with dampening measured via the more comprehensive LEDS, low pro-hedonic ER goal endorsement uniquely contributed to the prediction of concurrent dampening. Notably, the models including hedonic ER goal endorsement and NEC avoidance found support for the unique predictive validity of both factors.

Together, the results add to the recent cross-sectional evidence for the idea that dampening may be a strategy to avoid the experience of NECs (Malivoire et al. 2022). That is, a higher tendency toward NEC avoidance uniquely predicted a concurrent propensity to dampen PA, even after controlling for (the NEC avoidance function of) RNT. Our findings also suggest that the proposed NEC avoidance function of dampening can likely not be reduced to its presumed emotion preference-congruent ER function as such. Moreover, although fear of NA is frequently endorsed by those with depression (Yoon et al. 2018), the experience of a NEC (i.e., sharp decrease in PA or sharp increase in NA) might be viewed as even worse than NA per se. A recent momentary assessment study indeed showed that people suffering from MDD/GAD were more likely than those without MDD/GAD to repeatedly indicate that they intentionally focused on the negative to avoid NECs and felt greater vulnerability to NECs when feeling happiness (Baik and Newman 2023). Thus, dampening might be driven by NEC avoidance—extending the Contrast Avoidance Model (Newman and Llera 2011)—even at the cost of enhancing overall NA, as demonstrated by the experimental dampening induction studies (Burr et al. 2017; Dunn et al. 2018).

Beyond the possible NEC avoidance function of dampening, also contra-hedonic ER goal endorsement consistently contributed to the prediction of one’s tendency to dampen PA. This seemingly more prominent role of high contra-hedonic (vs. low pro-hedonic) ER goal endorsement dovetails with the Reward Devaluation Theory (RDT; Winer and Salem 2016). The RDT was introduced to explain the bias away from positive (vs. neutral) stimuli in individuals suffering from depression (Gallagher et al. 2023). It states that depressed individuals are not only characterized by the inhibition of PA approach behavior (cf. low pro-hedonic goal endorsement), but to an important extent also by active PA avoidance and PA devaluation (cf. high contra-hedonic goal endorsement; Gallagher et al. 2023). Although PA devaluation theoretically differs from dampening—since dampening does not imply that PA is processed as negative (Gallagher et al. 2023)—it may still reflect active PA avoidance rather than mere disengagement from PA approach behavior.

The present findings, that NEC avoidance and contra-hedonic goal endorsement were uniquely associated with concurrent dampening, provide a foundation for further research. For instance, future studies should investigate whether both tendencies underlie the engagement in dampening thoughts, parallelling research on the motivational component of the CAM for negative thinking (Baik and Newman 2023). Such future research could start with addressing the limitations of the current study. First, and most importantly, the current study relied on cross-sectional self-report methods. Future study designs yielding panel data and/or time series could ensure valid inferences about the directionality of the observed associations. An additional advantage of time series is the reduced risk of cognitive biases, such as a recall bias, and increased ecological validity (Myin-Germeys and Kuppens 2022).

Second, this study utilized Prolific as an online platform for data collection, which may introduce certain limitations regarding data quality and generalizability. Although Prolific generally provides relatively high data quality (Peer et al. 2022), total reaction times for survey completion were considered to verify data quality, and our findings were consistent with prior literature and theoretical expectations, future research may benefit from incorporating additional safeguards to minimize sources of potential confounding. For instance, attention checks or control questions could be included to objectively verify data quality (Tiersma et al. 2022). Generalizability to other, especially clinical samples, may be limited due to a potential selection bias. To address this, future studies could enhance representativeness of the study sample by integrating multiple recruitment strategies and employing stratified sampling methods. Minimally, future studies should assess the presence of symptoms of psychopathology known to be associated with dampening to enable a more detailed sample characterization and to better estimate its representativeness across clinical and nonclinical populations.

Third, the discomfort domain-specific factor of the CAQ-GE (Llera and Newman 2017) does not allow us to make inferences about positive emotional contrasts (PECs), or brief bouts of increased PA or reduced NA. One phenomenon worth mentioning here is the mood brightening effect, observed in depressed (vs. nondepressed) individuals. That is, depressed individuals’ mood brightened, or showed greater reductions in NA and/or increases in PA (vs. nondepressed people) in response to daily positive events (Bylsma et al. 2011; Khazanov and Ruscio 2016; Panaite et al. 2019; Peeters et al. 2003; Thompson et al. 2012). Some evidence in the anxiety and depression literature suggests that dampening may increase the likelihood of experiencing such PECs when encountering positive events or when a feared outcome does not happen (Baik & Newman 2025; Kim & Newman 2023; Llera & Newman 2017; Newman et al. 2019; Newman et al. 2022; Vîslă et al. 2021). Emerging evidence also suggests that individuals with depression or anxiety may make intentional, motivated use of dampening strategies to increase the likelihood of experiencing PECs. For example, Llera and Newman (2017) found that individuals with GAD scored significantly higher than controls on the “worry to create a positive contrast” subscale of the CAQ-W. In a more recent study, both higher baseline GAD and higher baseline MDD symptoms predicted higher ongoing momentary ratings on intentionally thinking pessimistically to be pleasantly surprised if something good happened (Baik & Newman 2025). Alternatively, it could simply be an unintended by-product of starting from a lower affective baseline, especially given positive emotion leaves individuals vulnerable to future NECs. In fact, Baik and Newman (2023), found that those with anxiety and/or depression were more likely to endorse ongoing “greater vulnerability to NECs when feeling positive.” Developing a general scale to distinguish between behaviors aimed at enhancing PECs versus minimizing NECs could be an important first step.

Relatedly, the pro- and contra-hedonic ER subscales of the Emotional Regulation Goals Scale (ERGS; Eldesouky and English 2019) conflate ER of positive and negative emotions. Although the two-factor structure was confirmed in the current study (i.e., pro- and contra-hedonic), future studies might want to disentangle this distinction (Bloore et al. 2020).

Finally, the current findings suggested that the presumed emotion preference-congruent ER function of dampening could not be reduced to the NEC avoidance function. Therefore, future studies should examine the relative importance of other proposed underlying motives of dampening which may explain the diminished preference for PA. For instance, the endorsement of contra-hedonic ER goals might also be an expression of an underlying self-verification goal. As stated by the Self-Verification theory (Swann et al. 1992), having one’s self-view verified is reinforcing (Brummelman et al. 2016; Swann 2012; Winer et al. 2011), and dampening may be a way to experience emotional states that are in line with one’s negative self-view (Vanderlind et al. 2020). Relatedly, it should be explicitly mentioned that gaining insight into the relative importance of motives underlying dampening requires individuals to have sufficient awareness and comprehension of their emotional lives, emotional preferences, and related ER goals.

Strong evidence establishing the directionality and causality of dampening’s presumed NEC avoidance function is needed. However, if confirmed, it may hold important clinical implications. First, interventions should help individuals to realize that the short-term benefits of habitual attempts to avoid sharp emotional shifts (Kim and Newman 2022; LaFreniere and Newman 2023a) via dampening (e.g., relief, sense of control) might come with severe long-term costs. Habitually downplaying PA might contribute to the chronicity of psychopathology.

In addition, individuals should be equipped with alternatives to counterbalance their seemingly natural attitude to avoid strong increases in NA and, consequently also PA experiences. One candidate may be strengthening decentering-related abilities (also called self-as-context or self-distancing; overview by Bennett et al. 2021; or cognitive defusion within acceptance based treatments; Hayes 2004). In essence, those abilities involve adopting an objective perspective and noticing (negative) inner events—such as the vulnerability to NECs—without reacting in an inappropriate way (Bernstein et al. 2015; Eftekhari et al. 2017) or becoming fused with them (Hayes 2004). Alternatively, also strengthening savoring may help individuals to build acceptance of NEC vulnerability (LaFreniere and Newman 2023a). Savoring is considered an adaptive response style that maintains, intensifies, or prolongs PA experiences via appreciating pleasurable life events (Bryant 1989; Bryant and Veroff 2007). Savoring thus inevitably results in exposure to affective fluctuations, including NECs when PA returns to one’s personal affective average. Recently, the effectiveness of an ecological momentary intervention was found to improve PA, optimism, and savoring and reduce dampening (LaFreniere and Newman 2023b; LaFreniere and Newman 2024). As the intentions to actively engage in a savoring or dampening are incompatible (LaFreniere and Newman 2023b; LaFreniere and Newman 2024), improving savoring might, in the long term, result in less dampening.

One probable pitfall of the training process toward alternative PA response styles may be the short-term increase of the tendency to dampen PA, as PA exposure increases. Initially, it may result in ambivalent experiences. Two promising strategies to overcome this, as adopted in Augmented Depression Therapy (ADepT; Dunn et al. 2023), are psycho-education to ensure individuals have realistic expectations of the training process (e.g., practicing savoring techniques involves dropping dampening as a safety behavior), and good self-care after positive experiences.

In sum, this study supports the possibility that dampening may be partly motivated by the avoidance of NECs, beyond mere emotional preferences. If further research confirms this presumed underlying mechanism of dampening, it could inform the development of more effective interventions targeting PA dysregulation across psychopathologies.

## Supplementary Material

supplement

Additional supporting information can be found online in the Supporting Information section.

Supplementary Material R FINAL.

## Figures and Tables

**TABLE 1 |:** Descriptive statistics.

	*M*	SD	Minimum	Maximum
RPA dampening subscale	15.53	4.62	8	30
LEDS	23.30	9.31	13	65
CAQ-GE discomfort emotional shifts	21.02	6.56	7	35
ERGS—pro-hedonic	12.69	3.50	4	21
ERGS—contra-hedonic	6.72	3.55	3	21
PTQ core characteristics (RNT)	21.07	7.93	0	36

*Note:* Descriptive statistics of reported levels of dampening measured via the RPA dampening subscale were almost identical to normative data (e.g., Feldman et al. 2008: *M* = 15.53, SD = 4.49); No formal normative data were available yet to compare descriptive statistics of the reported dampening levels via the LEDS; Descriptive statistics of reported levels of discomfort with emotional shifts fell between normative data of individuals of the “anxious” versus “non-anxious” group of the original validation study (Llera and Newman 2017); For the emotion regulation goals endorsement, descriptive statistics were highly similar to normative data (e.g., Eldesouky and English 2018; reported mean scores were transformed to sum scores, *M*_pro-hedonic_ = 13.38, *M*_contra-hedonic_ = 6.57); Normative data for the PTQ core characteristics (e.g., McEvoy et al. 2018: *M* = 25.33, SD = 7.84) slightly exceeded the reported levels of RNT in the current sample. However, this difference is likely attributable to the stratified sampling approach for gender adopted in the current study (i.e., 49% female vs. 61.1% female in the normative sample), as women generally report higher levels of RNT.

Abbreviations: CAQ-GE, Contrast Avoidance Questionnaire—General Emotion; ERGS, Emotion Regulation Goal Scale; LEDS, Leuven Exeter Dampening Scale; PTQ, Perseverative Thinking Questionnaire; RPA, Responses to Positive Affect Scale.

**TABLE 2 |:** Correlational matrix (Pearson’s correlations).

	1	2	3	4	5	6
1. RPA-d (dampening)	—					
2. LEDS (dampening)	0.75**	—				
3. CAQ-GE discomfort emotional shifts	0.40**	0.49**	—			
4. ERGS—pro-hedonic	0.12 (0.11)	0.04 (0.64)	0.37**	—		
5. ERGS—contra-hedonic	0.39**	0.52**	0.21*	0.11 (0.17)	—	
6. PTQ (RNT)	0.45**	0.48**	0.57**	0.23*	0.25*	—

*Note:* Values between brackets refer to insignificant *p* (> *α* = 0.05).

* *p* < 0.05;

** *p* < 0.001.

**TABLE 3 |:** Multiple linear regression models with and without considering the possibly confounding role of RNT.

	DV = dampening (RPA-d)								DV = dampening (LEDS)							
	B (SE)	*β*	*t*	*p* value	B (SE)	*β*	*t*	*p* value	B (SE)	*β*	*t*	*p* value	B (SE)	*β*	*t*	*p* value
	Model 1a F(3, 152) = 15.84, *p* < 0.001; *R*^2^ adjusted = 0.22				Model 1b F(2, 153) = 5.59, *p* = 0.005; *R*^2^ adjusted = 0.06				Model 1a F(3, 152) = 13.18, *p* < 0.001; *R*^2^ adjusted = 0.19				Model 1b F(2, 153) = 4.91, *p* = 0.009; *R*^2^ adjusted = 0.05			
Intercept	12.51 (1.38)	—	9.05	< 0.001**	18.48 (1.02)	—	18.13	< 0.001**	17.48 (2.75)	—	6.34		28.31 (2.00)	—	14.18	< 0.001**
Gender	−1.42 (0.61)	−0.17	−2.34	0.02*	−0.86 (0.66)	−0.10	−1.29	0.20	−0.99 (1.20)	−0.06	−0.82	< 0.001**	0.02 (1.29)	0.001	0.02	0.99
Age	−0.04 (0.02)	−0.12	−1.64	0.10	−0.08 (0.03)	−0.24	−3.04	0.003*	−0.09 (0.05)	−0.14	−1.82	0.41 0.07	−0.16 (0.05)	−0.25	−3.13	0.002*
RNT	0.23 (0.04)	0.43	5.83	< 0.001**	—	—	—	—	0.43 (0.08)	0.40	5.29	< 0.001**	—	—	—	—
	Model 2a F(4, 151) = 13.70, *p* < 0.001; *R*^2^ adjusted = 0.25^Δ*R*^2^^ = 0.024 (*f*^2^ = 0.04)				Model 2b F(3, 152) = 12.33, *p* < 0.001; *R*^2^ adjusted = 0.18; Δ*R*^2^ = 0.13 (*f*^2^ = 0.16)				Model 2a F(4, 151) = 15.02, *p* < 0.001; *R*^2^ adjusted = 0.37; Δ*R*^2^ =0.08 (*f*^2^ = 0.11)				Model 2b F(3, 152) = 16.91, *p* < 0.001; *R*^2^ adjusted = 0.24; Δ*R*^2^ = 0.19 (*f*^2^ = 0.23)			
Intercept	10.74 (1.55)	—	6.95	< 0.001**	12.58 (1.53)	—	8.21	< 0.001**	11.82 (2.97)	—	3.98**	< 0.001**	14.42 (2.87)	—	5.03	< 0.001**
Gender	−1.68 (0.61)	−0.20	−2.76	0.007*	−1.58 (0.64)	−0.19	−2.48	01*	−1.84 (1.16)	−0.11	−1.58 (0.12)	0.12	−1.71 (1.19)	−0.10	−1.44	0.15
Age	−0.03 (0.02)	−0.03	−1.33	0.18	−0.05 (0.02)	−0.14	−1.90	0.06	−0.06 (0.05)	−0.10	−1.39 (0.17)	0.17	−0.08 (0.05)	−0.13	−1.83	0.07
RNT	0.18 (0.05)	0.33	3.81	< 0.001**	—	—	—	—	0.24 (0.09)	0.09	2.69**	0.008*	—	—	—	—
NEC avoidance	0.14 (0.06)	0.21	2.40	0.02*	0.25 (0.05)	0.38	4.91	< 0.001**	0.44 (0.11)	0.34	4.06**	< 0.001**	0.58 (0.09)	0.46	6.21	< 0.001**
	Model 3a F(6, 149) = 12.06, *p* < 0.001; *R*^2^ adjusted = 0.30; Δ*R*^2^ = 0.053 (*f*^2^ contra-hedonic = 0.08; *f*^2^ pro-hedonic = 0.002)				Model 3b F(5, 150) = 11.26, *p* < 0.001; *R*^2^ adjusted = 0.25; Δ*R*^2^ = 0.07 (*f*^2^ contra-hedonic = 0.10; *f*^2^ pro-hedonic = 0.002)				Model 3a F(6, 149) = 19.19, *p* < 0.001; *R*^2^ adjusted = 0.41; Δ*R*^2^ = 0.15 (*f*^2^ contra-hedonic = 0.24; *f*^2^ pro-hedonic = 0.03)				Model 3b F(5,150) = 21.34, *p* < 0.001; *R*^2^ adjusted = 0.40; Δ*R*^2^ = 0.16 (*f*^2^ contra-hedonic = 0.26; *f*^2^ pro-hedonic = 0.03)			
Intercept	8.45 (1.83)	—	4.61	< 0.001**	9.77 (1.86)	—	5.26	< 0.001**	9.37 (3.27)	—	2.87	0.004*	11.00 (3.23)	—	3.40	< 0.001**
Gender	−1.47 (0.59)	−0.17	−2.48	0.01*	−1.35 (0.61)	−0.16	0.61	0.03*	−1.19 (1.05)	−0.07	−1.14	0.26	−1.07 (1.06)	−0.07	−1.01	0.32
Age	−0.01 (0.02)	−0.04	−0.58	0.56	−0.02 (0.02)	−0.07	−1.00	0.32	−0.03 (0.04)	−0.05	−0.73	0.46	−0.04 (0.04)	−0.07	−1.05	0.30
RNT	0.16 (0.04)	0.29	3.46	< 0.001**	—	—	—	—	0.19 (0.08)	0.18	2.31	0.02*	—	—	—	—
NEC avoidance	0.12 (0.06)	0.18	2.03	0.04*	0.21 (0.05)	0.32	4.05	< 0.001**	0.46 (0.10)	0.36	4.60	< 0.001**	0.57 (0.09)	0.45	6.38	< 0.001**
Contra-hedonic ER	0.31 (0.09)	0.26	3.65	< 0.001*	0.35 (0.09)	0.29	3.97	< 0.001**	0.92 (0.15)	0.39	5.96	< 0.001**	0.96 (0.16)	0.40	6.19	< 0.001**
Pro-hedonic ER	0.02 (0.09)	0.02	0.26	0.80	0.03 (0.09)	0.02	0.28	0.78				−0.36 (0.16)	−0.15	−2.21	0.03*	−0.34 (0.16)
−0.15	−2.14	0.03*														

*Note:* Δ*R*^2^ refers to *R*^2^ change compared with the model in the previous step.

* *p* < 0.05;

** *p* < .001.

## Data Availability

The data that support the findings of this study are openly available in OSF at https://osf.io/23sgf/. The anonymised data and analysis code are publicly available on https://osf.io/23sgf/.

## References

[R1] Abasi I, Shams G, Pascual-Vera B, 2023. “Positive Emotion Regulation Strategies as Mediators in Depression and Generalized Anxiety Disorder Symptoms: A Transdiagnostic Framework Investigation.” Current Psychology 42, no. 1: 800–807. 10.1007/s12144-021-01392-5.

[R2] American Psychiatric Association. 2013. “Diagnostic and Statistical Manual of Mental Disorders (DSM-5; 5th ed.).” American Psychiatric Association. 10.1176/appi.books.9780890425596.

[R3] Baik SY, and Newman MG. 2023. “The Transdiagnostic Use of Worry and Rumination to Avoid Negative Emotional Contrasts Following Negative Events: A Momentary Assessment Study.” Journal of Anxiety Disorders 95: 102679. 10.1016/j.janxdis.2023.102679. 36863193 PMC10191629

[R4] Baik SY, and Newman MG. 2025. “Why Do Individuals With Generalized Anxiety Disorder and Depression Engage in Worry and Rumination?: A Momentary Assessment Study of Positive Contrast Enhancement.” Journal of Anxiety Disorders 111: 102982. 10.1016/j.janxdis.2025.102982. 39947018 PMC12054337

[R5] Bean CAL, Summers CB, and Ciesla JA. 2022. “Dampening of Positive Affect and Depression: A Meta-Analysis of Cross-Sectional and Longitudinal Relationships.” Behaviour Research and Therapy 156: 104153. 10.1016/j.brat.2022.104153. 35863241

[R6] Bennett MP, Knight R, Patel S, 2021. “Decentering as a Core Component in the Psychological Treatment and Prevention of Youth Anxiety and Depression: A Narrative Review and Insight Report.” Translational Psychiatry 11, no. 1: 288. 10.1038/s41398-021-01397-5. 33990541 PMC8121888

[R7] Bernstein A, Hadash Y, Lichtash Y, Tanay G, Shepherd K, and Fresco DM. 2015. “Decentering and Related Constructs: A Critical Review and Metacognitive Processes Model.” Perspectives on Psychological Science 10, no. 5: 599–617. 10.1177/1745691615594577. 26385999 PMC5103165

[R8] Bloore RA, Jose PE, and Roseman IJ. 2020. “General Emotion Regulation Measure (GERM): Individual Differences In Motives of Trying to Experience and Trying to Avoid Experiencing Positive and Negative Emotions.” Personality and Individual Differences 166: 110174. 10.1016/j.paid.2020.110174.

[R9] Boelen PA 2021. “Dampening of Positive Affect Is Associated With Posttraumatic Stress Following Stressful Life Events.” European Journal of Psychotraumatology 12, no. 1: 1851077. 10.1080/20008198.2020.1851077. 33505637 PMC7817208

[R10] Bogaert L, Lau H, Dunn BD, and Raes F. 2025. “The Leuven Exeter Dampening Scale (LEDS) to Measure Dampening Appraisals Towards Positive Affect: Psychometric Evaluation and Initial Validation in a Dutch and English Community Sample.” Behaviour Research and Therapy 194: 104861. 10.1016/j.brat.2025.104861. 41067132

[R11] Brummelman E, Crocker J, and Bushman BJ. 2016. “The Praise Paradox: When and Why Praise Backfires in Children With Low Self-Esteem.” Child Development Perspectives 10, no. 2: 111–115. 10.1111/cdep.12171.

[R12] Bryant FB 1989. “A Four-Factor Model of Perceived Control: Avoiding, Coping, Obtaining, and Savoring.” Journal of Personality 57, no. 4: 773–797. 10.1111/j.1467-6494.1989.tb00494.x.

[R13] Bryant FB, and Veroff J. 2007. Savoring: A New Model of Positive Experience (1st ed. Psychology Press). 10.4324/9781315088426.

[R14] Buhk AH, Schadegg MJ, Dixon LJ, and Tull MT. 2020. “Investigating the Role of Negative and Positive Emotional Avoidance in the Relation Between Generalized Anxiety Disorder and Depression Symptom Severity.” Journal of Contextual Behavioral Science 16: 103–108. 10.1016/j.jcbs.2020.03.006.

[R15] Burr LA, Javiad M, Jell G, Werner-Seidler A, and Dunn BD. 2017. “Turning Lemonade Into Lemons: Dampening Appraisals Reduce Positive Affect and Increase Negative Affect During Positive Activity Scheduling.” Behaviour Research and Therapy 91: 91–101. 10.1016/j.brat.2017.01.010. 28167331

[R16] Bylsma LM, Taylor-Clift A, and Rottenberg J. 2011. “Emotional Reactivity to Daily Events in Major and Minor Depression.” Journal of Abnormal Psychology 120, no. 1: 155–167. 10.1037/a0021662. 21319928

[R17] Damme K, Gupta T, Haase CM, and Mittal VA. 2022. “Responses to Positive Affect and Unique Resting-State Connectivity in Individuals at Clinical High-Risk for Psychosis.” NeuroImage. Clinical 33: 102946. 10.1016/j.nicl.2022.102946. 35091254 PMC8800133

[R18] Dunn BD, and Roberts H. 2016. “Improving the Capacity to Treat Depression Using Talking Therapies.” In The Wiley Handbook of Positive Clinical Psychology, edited by In. Wood AM and Johnson J, 181–204. Wiley. 10.1002/9781118468197.ch13.

[R19] Dunn BD, Widnall E, Warbrick L, 2023. “Preliminary Clinical and Cost Effectiveness of Augmented Depression Therapy Versus Cognitive Behavioural Therapy for the Treatment of Anhedonic Depression (ADepT): A Single-Centre, Open-Label, Parallel-Group, Pilot, Randomised, Controlled Trial.” EClinicalMedicine 61: 102084. 10.1016/j.eclinm.2023.102084. 37528846 PMC10388573

[R20] Dunn BD, Burr LA, Smith HB, 2018. “Turning Gold Into Lead: Dampening Appraisals Reduce Happiness and Pleasantness and Increase Sadness During Anticipation and Recall of Pleasant Activities in the Laboratory.” Behaviour Research and Therapy 107: 19–33. 10.1016/j.brat.2018.05.003. 29807349

[R21] Dworschak C, Polack RG, Winschel J, Joormann J, and Kober H. 2023. “Emotion Regulation and Disordered Eating Behaviour in Youths: Two Daily-Diary Studies.” European Eating Disorders Review 31, no. 5: 655–669. 10.1002/erv.2993. 37293697

[R22] Eftekhari E, Tran A, and McGregor I. 2017. “Decentering Increases Approach Motivation Among Distressed Individuals.” Personality and Individual Differences 119: 236–241. 10.1016/j.paid.2017.07.035.

[R23] Ehring T, Zetsche U, Weidacker K, Wahl K, Schönfeld S, and Ehlers A. 2011. “The Perseverative Thinking Questionnaire (PTQ): Validation of a Content-Independent Measure of Repetitive Negative Thinking.” Journal of Behavior Therapy and Experimental Psychiatry 42, no. 2: 225–232. 10.1016/j.jbtep.2010.12.003. 21315886 PMC3042595

[R24] Eisner LR, Johnson SL, and Carver CS. 2009. “Positive Affect Regulation in Anxiety Disorders.” Journal of Anxiety Disorders 23, no. 5: 645–649. 10.1016/j.janxdis.2009.02.001. 19278820 PMC2847490

[R25] Eldesouky L, and English T. 2018. “Another Year Older, Another Year Wiser? Emotion Regulation Strategy Selection and Flexibility Across Adulthood.” Psychology and Aging 33, no. 4: 572–585. 10.1037/pag0000251. 29745687 PMC6002946

[R26] Eldesouky L, and English T. 2019. “Individual Differences in Emotion Regulation Goals: Does Personality Predict the Reasons Why People Regulate Their Emotions?” Journal of Personality 87, no. 4: 750–766. 10.1111/jopy.12430. 30246480 PMC6431278

[R27] Everaert J, Benisty H, Gadassi Polack R, Joormann J, and Mishne G. 2022. “Which Features of Repetitive Negative Thinking and Positive Reappraisal Predict Depression? An In-Depth Investigation Using Artificial Neural Networks With Feature Selection.” Journal of Psychopathology and Clinical Science 131, no. 7: 754–768. 10.1037/abn0000775. 35862088

[R28] Everaert J, Bronstein MV, Castro AA, Cannon TD, and Joormann J. 2020. “When Negative Interpretations Persist, Positive Emotions Don’t! Inflexible Negative Interpretations Encourage Depression and Social Anxiety by Dampening Positive Emotions.” Behaviour Research and Therapy 124: 103510. 10.1016/j.brat.2019.103510. 31734567

[R29] Feldman GC, Joormann J, and Johnson SL. 2008. “Responses to Positive Affect: A Self-Report Measure of Rumination and Dampening.” Cognitive Therapy and Research 32, no. 4: 507–525. 10.1007/s10608-006-9083-0. 20360998 PMC2847784

[R30] Forbes EE, Olino TM, Ryan ND, 2010. “Reward-Related Brain Function as a Predictor of Treatment Response in Adolescents With Major Depressive Disorder.” Cognitive, Affective, & Behavioral Neuroscience 10, no. 1: 107–118. 10.3758/CABN.10.1.107. PMC2841787 20233959

[R31] Gallagher MR, Collins AC, and Winer ES. 2023. “A Network Analytic Investigation of Avoidance, Dampening, and Devaluation of Positivity.” Journal of Behavior Therapy and Experimental Psychiatry 81: 101870. 10.1016/j.jbtep.2023.101870. 37201468 PMC10524699

[R32] Hayes SC 2004. “Acceptance and Commitment Therapy, Relational Frame Theory, and the Third Wave of Behavioral and Cognitive Therapies.” Behavior Therapy 35, no. 4: 639–665. 10.1016/S0005-7894(04)80013-3. 27993338

[R33] Jamil N, and Llera SJ. 2021. “A Transdiagnostic Application of the Contrast-Avoidance Model: The Effects of Worry and Rumination in a Personal-Failure Paradigm.” Clinical Psychological Science 9, no. 5: 836–849. 10.1177/2167702621991797.

[R34] Joormann J, and Stanton CH. 2016. “Examining Emotion Regulation in Depression: A Review and Future Directions.” Behaviour Research and Therapy 86: 35–49. 10.1016/j.brat.2016.07.007. 27492851

[R35] Joormann J, and Gotlib IH. 2010. “Emotion Regulation in Depression: Relation to Cognitive Inhibition.” Cognition & Emotion 24, no. 2: 281–298. 10.1080/02699930903407948. 20300538 PMC2839199

[R36] Joormann J, and Vanderlind WM. 2014. “Emotion Regulation in Depression: The Role of Biased Cognition and Reduced Cognitive Control.” Clinical Psychological Science 2, no. 4: 402–421. 10.1177/2167702614536163.

[R37] Kendall AD, Zinbarg RE, Mineka S, 2015. “Prospective Associations of Low Positive Emotionality With First Onsets of Depressive and Anxiety Disorders: Results From a 10-Wave Latent Trait-State Modeling Study.” Journal of Abnormal Psychology 124, no. 4: 933–943. 10.1037/abn0000105. 26372005 PMC4658315

[R38] Khazanov GK, and Ruscio AM. 2016. “Is Low Positive Emotionality a Specific Risk Factor for Depression? A Meta-Analysis of Longitudinal Studies.” Psychological Bulletin 142, no. 9: 991–1015. 10.1037/bul0000059. 27416140 PMC5110375

[R39] Kiefer R, Goncharenko S, Forkus SR, Contractor AA, LeBlanc N, and Weiss NH. 2023. “Role of Positive Emotion Regulation Strategies in the Association Between Childhood Trauma and Posttraumatic Stress Disorder Among Trauma-Exposed Individuals Who Use Substances.” Anxiety, Stress, & Coping 36, no. 3: 366–381. 10.1080/10615806.2022.2079636. 35603928 PMC9679041

[R40] Kim H, and Newman MG. 2022. “Avoidance of Negative Emotional Contrast From Worry and Rumination: An Application of the Contrast Avoidance Model.” Journal of Behavioral and Cognitive Therapy 32, no. 1: 33–43. 10.1016/j.jbct.2021.12.007. 35693377 PMC9181176

[R41] Kim H, and Newman MG. 2023. “Worry and Rumination Enhance a Positive Emotional Contrast Based on the Framework of the Contrast Avoidance Model.” Journal of Anxiety Disorders 94: 102671. 10.1016/j.janxdis.2023.102671. 36681058 PMC10071830

[R42] LaFreniere LS, and Newman MG. 2023a. “Reducing Contrast Avoidance in GAD by Savoring Positive Emotions: Outcome and Mediation in a Randomized Controlled Trial.” Journal of Anxiety Disorders 93: 102659. 10.1016/j.janxdis.2022.102659. 36549218 PMC9976801

[R43] LaFreniere LS, and Newman MG. 2023b. “Upregulating Positive Emotion in Generalized Anxiety Disorder: A Randomized Controlled Trial of the Skilljoy Ecological Momentary Intervention.” Journal of Consulting and Clinical Psychology 91, no. 6: 381–387. 10.1037/ccp0000794. 36716146 PMC10580378

[R44] LaFreniere LS, and Newman MG. 2024. “Savoring Changes Novel Positive Mindset Targets of GAD Treatment: Optimism, Prioritizing Positivity, Kill-Joy Thinking, and Worry Mediation.” Behaviour Research and Therapy 177: 104541. 10.1016/j.brat.2024.104541. 38640622 PMC11096009

[R45] Lenferink LIM, Wessel I, and Boelen PA. 2018. “Exploration of the Associations Between Responses to Affective States and Psychopathology in Two Samples of People Confronted With the Loss of a Loved One.” Journal of Nervous & Mental Disease 206, no. 2: 108–115. 10.1097/NMD.0000000000000781. 29293167

[R46] Li YI, Starr LR, and Hershenberg R. 2017. “Responses to Positive Affect in Daily Life: Positive Rumination and Dampening Moderate the Association Between Daily Events and Depressive Symptoms.” Journal of Psychopathology and Behavioral Assessment 39, no. 3: 412–425. 10.1007/s10862-017-9593-y.

[R47] Llera SJ, and Newman MG. 2017. “Development and Validation of Two Measures of Emotional Contrast Avoidance: The Contrast Avoidance Questionnaires.” Journal of Anxiety Disorders 49: 114–127. 10.1016/j.janxdis.2017.04.008. 28500921 PMC8765496

[R48] Lüdecke D, Ben-Shachar M, Patil I, Waggoner P, and Makowski D. 2021. “Performance: An R Package for Assessment, Comparison and Testing of Statistical Models.” Journal of Open Source Software 6, no. 60: 3139. 10.21105/joss.03139.

[R49] Malivoire BL, Marcotte-Beaumier G, Sumantry D, and Koerner N. 2022. “Correlates of Dampening and Savoring in Generalized Anxiety Disorder.” International Journal of Cognitive Therapy 15, no. 4: 414–433. 10.1007/s41811-022-00145-x. 36161248 PMC9483300

[R50] McEvoy PM, Hyett MP, Ehring T, 2018. “Transdiagnostic Assessment of Repetitive Negative Thinking and Responses to Positive Affect: Structure and Predictive Utility for Depression, Anxiety, and Mania Symptoms.” Journal of Affective Disorders 232: 375–384. 10.1016/j.jad.2018.02.072. 29510356

[R51] Millgram Y, Joormann J, Huppert JD, Lampert A, and Tamir M. 2019. “Motivations to Experience Happiness or Sadness in Depression: Temporal Stability and Implications for Coping With Stress.” Clinical Psychological Science 7, no. 1: 143–161. 10.1177/2167702618797937.

[R52] Morris BH, Bylsma LM, and Rottenberg J. 2009. “Does Emotion Predict the Course of Major Depressive Disorder? A Review of Prospective Studies.” British Journal of Clinical Psychology 48, no. Pt 3: 255–273. 10.1348/014466508X396549. 19187578

[R53] Moulds ML, and McEvoy PM. 2025. “Repetitive Negative Thinking as a Transdiagnostic Cognitive Process.” Nature Reviews Psychology 4, no. 2: 127–141. 10.1038/s44159-024-00399-6.

[R54] Myin-Germeys I, and Kuppens P, eds. 2022. The Open Handbook of Experience Sampling Methodology: A Step-By-Step Guide to Designing, Conducting, and Analyzing ESM Studies. 2nd ed., 7–19. Center for Research on Experience Sampling and Ambulatory Methods Leuven.

[R55] Newman MG, and Llera SJ. 2011. “A Novel Theory of Experiential Avoidance in Generalized Anxiety Disorder: A Review and Synthesis of Research Supporting a Contrast Avoidance Model of Worry.” Clinical Psychology Review 31, no. 3: 371–382. 10.1016/j.cpr.2011.01.008. 21334285 PMC3073849

[R56] Newman MG, Rackoff GN, Zhu Y, and Kim H. 2023. “A Transdiagnostic Evaluation of Contrast Avoidance Across Generalized Anxiety Disorder, Major Depressive Disorder, and Social Anxiety Disorder.” Journal of Anxiety Disorders 93: 102662. 10.1016/j.janxdis.2022.102662. 36565682 PMC10080671

[R57] Newman MG, Schwob JT, Rackoff GN, Doren NV, Shin KE, and Kim H. 2022. “The Naturalistic Reinforcement of Worry From Positive and Negative Emotional Contrasts: Results From a Momentary Assessment Study Within Social Interactions.” Journal of Anxiety Disorders 92: 102634. 10.1016/j.janxdis.2022.102634. 36182690 PMC10187062

[R58] Newman MG, Jacobson NC, Zainal NH, Shin KE, Szkodny LE, and Sliwinski MJ. 2019. “The Effects of Worry in Daily Life: An Ecological Momentary Assessment Study Supporting the Tenets of the Contrast Avoidance Model.” Clinical Psychological Science 7, no. 4: 794–810. 10.1177/2167702619827019. 31372313 PMC6675025

[R59] Newman MG, Llera SJ, Erickson TM, Przeworski A, and Castonguay LG. 2013. “Worry and Generalized Anxiety Disorder: A Review and Theoretical Synthesis of Evidence on Nature, Etiology, Mechanisms, and Treatment.” Annual Review of Clinical Psychology 9, no. 1: 275–297. 10.1146/annurev-clinpsy-050212-185544. PMC4964851 23537486

[R60] Panaite V, Koval P, Dejonckheere E, and Kuppens P. 2019. “Emotion Regulation and Mood Brightening in Daily Life Vary With Depressive Symptom Levels.” Cognition and Emotion 33, no. 6: 1291–1301. 10.1080/02699931.2018.1543180. 30497322

[R61] Peer E, Rothschild D, Gordon A, Evernden Z, and Damer E. 2022. “Data Quality of Platforms and Panels for Online Behavioral Research.” Behavior Research Methods 54, no. 4: 1643–1662. 10.3758/s13428-021-01694-3. 34590289 PMC8480459

[R62] Peeters F, Nicolson NA, Berkhof J, Delespaul P, and deVries M. 2003. “Effects of Daily Events on Mood States in Major Depressive Disorder.” Journal of Abnormal Psychology 112, no. 2: 203–211. 10.1037/0021-843x.112.2.203. 12784829

[R63] Rackoff GN, and Newman MG. 2020. “Reduced Positive Affect on Days With Stress Exposure Predicts Depression, Anxiety Disorders, and Low Trait Positive Affect 7 Years Later.” Journal of Abnormal Psychology 129, no. 8: 799–809. 10.1037/abn0000639. 32914995 PMC8048702

[R64] Raes F, Smets J, Wessel I, 2014. “Turning the Pink Cloud Grey: Dampening of Positive Affect Predicts Postpartum Depressive Symptoms.” Journal of Psychosomatic Research 77, no. 1: 64–69. 10.1016/j.jpsychores.2014.04.003. 24913344

[R65] Raes F, Smets J, Nelis S, and Schoofs H. 2012. “Dampening of Positive Affect Prospectively Predicts Depressive Symptoms in Non-Clinical Samples.” Cognition & Emotion 26, no. 1: 75–82. 10.1080/02699931.2011.555474. 21756217

[R66] Raîche G, Walls TA, Magis D, Riopel M, and Blais JG. 2013. “Non-Graphical Solutions for Cattell’s Scree Test.” Methodology-European Journal of Research Methods for the Behavioral and Social Sciences 9, no. 1: 23–29. 10.1027/1614-2241/a000051.

[R67] Rogers TA, Gorday JY, Bardeen JR, and Benfer N. 2023. “Examining the Factor Structure and Incremental Utility of the Contrast Avoidance Questionnaires via Bifactor Analysis.” Journal of Personality Assessment 105, no. 2: 238–248. 10.1080/00223891.2022.2081921. 35674446

[R68] Stefanovic M, Rosenkranz T, Ehring T, Watkins ER, and Takano K. 2022. “Is a High Association Between Repetitive Negative Thinking and Negative Affect Predictive of Depressive Symptoms? A Clustering Approach for Experience-Sampling Data.” Clinical Psychological Science 10, no. 1: 74–89. 10.1177/21677026211009495.

[R69] Swann WB 2012. “Self-Verification Theory.” In Handbook of Theories of Social Psychology (Vol. 2), edited by Lange PAMV, Kruglanski AW, and Higgins ET, 23–42. Sage Publications. 10.4135/9781446249222.

[R70] Swann WB Jr., Stein-Seroussi A, and Giesler RB 1992. “Why People Self-Verify.” Journal of Personality and Social Psychology 62, no. 3: 392–401. 10.1037//0022-3514.62.3.392. 1560335

[R71] Tamir M 2009. “What Do People Want to Feel and Why?” Current Directions in Psychological Science 18, no. 2: 101–105. 10.1111/j.1467-8721.2009.01617.x.

[R72] Tamir M 2016. “Why Do People Regulate Their Emotions? A Taxonomy of Motives in Emotion Regulation.” Personality and Social Psychology Review 20, no. 3: 199–222. 10.1177/1088868315586325. 26015392

[R73] Thompson RJ, Mata J, Jaeggi SM, Buschkuehl M, Jonides J, and Gotlib IH. 2012. “The Everyday Emotional Experience of Adults With Major Depressive Disorder: Examining Emotional Instability, Inertia, and Reactivity.” Journal of Abnormal Psychology 121, no. 4: 819–829. 10.1037/a0027978. 22708886 PMC3624976

[R74] Tiersma K, Reichman M, Popok PJ, 2022. “The Strategies for Quantitative and Qualitative Remote Data Collection: Lessons From the COVID-19 Pandemic.” JMIR Formative Research 6, no. 4: e30055. 10.2196/30055. 35394441 PMC9034421

[R75] Vanderlind WM, Everaert J, and Joormann J. 2022. “Positive Emotion in Daily Life: Emotion Regulation and Depression.” Emotion (Washington, D.C.) 22, no. 7: 1614–1624. 10.1037/emo0000944. 33661667

[R76] Vanderlind WM, Millgram Y, Baskin-Sommers AR, Clark MS, and Joormann J. 2020. “Understanding Positive Emotion Deficits in Depression: From Emotion Preferences to Emotion Regulation.” Clinical Psychology Review 76: 101826. 10.1016/j.cpr.2020.101826. 32058881

[R77] Vîslă A, Zinbarg R, Hilpert P, Allemand M, and Flückiger C. 2021. “Worry and Positive Episodes in the Daily Lives of Individuals With Generalized Anxiety Disorder: An Ecological Momentary Assessment Study.” Frontiers in Psychology 12: 722881. 10.3389/fpsyg.2021.722881. 34777100 PMC8579489

[R78] Winer ES, and Salem T. 2016. “Reward Devaluation: Dot-Probe Meta-Analytic Evidence of Avoidance of Positive Information in Depressed Persons.” Psychological Bulletin 142, no. 1: 18–78. 10.1037/bul0000022. 26619211 PMC4688138

[R79] Winer ES, Cervone D, Newman LS, and Snodgrass M. 2011. “Subchance Perception: Anxious, Non-Defensive Individuals Identify Subliminally-Presented Positive Words at Below-Chance Levels.” Personality and Individual Differences 51, no. 8: 996–1001. 10.1016/j.paid.2011.08.010.

[R80] Wolkenstein L, Sommerhoff A, and Voss M. 2022. “Positive Emotion Dysregulation in Posttraumatic Stress Disorder.” Journal of Anxiety Disorders 86: 102534. 10.1016/j.janxdis.2022.102534. 35114432

[R81] Yilmaz M, Psychogiou L, Javaid M, Ford T, and Dunn BD. 2019. “Making the Worst of a Good Job: Induced Dampening Appraisals Blunt Happiness and n Adolescents During Pleasant Memory Recall.” Behaviour Research and Therapy 122: 103476. 10.1016/j.brat.2019.103476. 31539833

[R82] Yoon S, Dang V, Mertz J, and Rottenberg J. 2018. “Are Attitudes Towards Emotions Associated With Depression? A Conceptual and Meta-Analytic Review.” Journal of Affective Disorders 232: 329–340. 10.1016/j.jad.2018.02.009. 29510350

