# Supplementary material for: Dampening of Positive Affect Serves an Emotional Contrast Avoidance Function: Preliminary Evidence From an Adult Community Sample
Source: J Clin Psychol. Author manuscript; Available in PMC 2026 Jan 10. (PMC12788394; doi:10.1002/jclp.70060)
Supplement: supplement [file NIHMS2123450-supplement-supplement.docx]

**Supplementary Material**

**Table S1**

*Items of the Experimental Leuven Exeter Dampening Scale (LEDS, Bogaert et al., 2025), Accompanied With the Class of Dampening Appraisals Each Item Represents*

Instructions | People think and do many different things when they start to feel happy. Please indicate for each of the following items whether you (almost) never, sometimes, regularly, often or (almost) always think or do what is described in each statement each time when you start to feel happy, excited or enthusiastic. Please indicate what you generally do, not what you think you should do.

| 1 | 2 | 3 | 4 | 5 |
| --- | --- | --- | --- | --- |
| Almost never | Sometimes | Regularly | Often | Almost always |

When you start to feel happy, how often do you think …

| LEDS Item | Class of Commonly Observed Dampening Thoughts |
| --- | --- |
| 1. ‘If I show that I’m doing better now, then others will expect that I will always feel good from now on.’ | social appropriateness |
| 1. ‘I would have enjoyed this more previously.’ | unhelpful comparison to past experiences |
| 1. ‘Bad things will follow if I enjoy this.’ | prospective cognition (magical beliefs) |
| 1. ‘I’m not worthy of feeling good.’ | deservedness |
| 1. ‘Other people are enjoying it more.’ | unhelpful comparison to others’ experiences |
| 1. ‘I can’t enjoy this feeling, because I already have to look ahead to avoid (bad) surprises.’ | prospective cognition (intolerance of uncertainty) |
| 1. ‘This isn’t as good as I hoped.’ | unhelpful comparison to expected experiences |
| 1. ‘I’m only allowed to be happy when others are too.’ | responsibility/consideration |
| 1. ‘Something might happen at any time and I could easily lose my happiness.’ | prospective cognition (fragility of happiness) |
| 1. ‘This positive feeling isn’t real.’ | trust/belief |
| 1. ‘I prefer not to feel too hopeful and relaxed, to minimize the pain of disappointment when positive feelings have gone again.’ | prospective cognition (contrast effect) |
| 1. ‘This positive event is an exception that won’t be repeated.’ | minimizing/dismissing |
| 1. ‘There is no point to feeling good.’ | utility |

**Measures**

Model fit of the Leuven Exeter Dampening Scale (LEDS; Bogaert et al., 2025) was assessed using descriptive fit indices, including the root mean square error of approximation (RMSEA), the comparative fit index (CFI), and the standardized root mean square residual (SRMR). In line with established guidelines, RMSEA values below .06 and CFI values above .90 were interpreted as indicators of good model fit. SRMR values below .10 were taken to indicate acceptable fit, with values under .05 reflecting a good fit (Hu & Bentler, 1999). Collectively, the model fit indices from the confirmatory factor analysis, conducted via the “lavaan” R package (Rosseel, 2012), yielded support for the proposed one-factor solution (robust CFI = .91, robust RMSEA = .03, 90% CI [.01;.04], SRMR = .06).

Parallel analysis (Raîche et al., 2013) and subsequent exploratory factor analysis on the items of the hedonic subscales of the Emotional Regulation Goals Scale (ERGS; Eldesouky & English, 2018) supported the two-factor structure. Details of the exploratory factor analysis can be found in Table S2.

**Table S2**

*Exploratory Factor Analysis: Factor Loadings and Explained Variance by Two Latent Factors (Oblimin Rotation)*

| Item | Factor 1  (Contra-Hedonic) | Factor 2  (Pro-Hedonic) | Complexity | Uniqueness |
| --- | --- | --- | --- | --- |
| Item 4 (To feel more negative emotion) | .93 |  | 1.00 | 0.14 |
| Item 6 (To keep feeling negative emotion) | .68 |  | 1.00 | 0.54 |
| Item 5 (To feel less positive emotion) | .60 |  | 1.06 | 0.62 |
| Item 2 (To feel more positive emotion) |  | .88 | 1.00 | 0.23 |
| Item 3 (To keep feeling positive emotion) |  | .80 | 1.00 | 0.36 |
| Item 1 (To feel less negative emotion) |  | .52 | 1.00 | 0.74 |
| % total variance explained | 56.37% | 28.22% |  |  |

**References**

Hu, L., & Bentler, P. M. (1999). Cutoff criteria for fit indexes in covariance structure analysis: Conventional criteria versus new alternatives. *Structural Equation Modeling: A Multidisciplinary Journal, 6*(1), 1–55. https://doi.org/10.1080/10705519909540118

Raîche, G., Walls, T. A., Magis, D., Riopel, M., & Blais, J.-G. (2013). Non-graphical solutions for Cattell’s scree test. *Methodology: European Journal of Research Methods for the Behavioral and Social Sciences, 9*(1), 23–29. <https://doi.org/f4qn7z>

Rosseel, Y. (2012). lavaan: An R package for structural equation modeling. *Journal of Statistical Software, 48*(2), 1–36. https://doi.org/10.18637/jss.v048.i02
